# Supplementary material for: Are carbon emissions trading and green financial instruments synergistic? -Comprehensive quantitative research based on content analysis
Source: PLoS One. 2024 Mar 7;19(3):e0298601. doi: 10.1371/journal.pone.0298601 (PMC10919601; doi:10.1371/journal.pone.0298601)
Supplement: S1 Data — (ZIP) [file pone.0298601.s001.zip › Data/地级市Z_F工作报告.zip!/╕╩╦α/╜≡▓2╩╨/└┤╫╘╣1⁄2╓┌║┼í╛┬φ┐╦╩2╛▌═°í┐_2015.docx]

各位代表：

现在，我代表市人民政府向大会作工作报告，请予审议，并请各位政协委员和列席会议的同志提出意见。

　　一、2014年工作回顾

　　刚刚过去的一年，面对经济下行压力加大、工业产品价格低迷等不利因素和各种困难，我们在市委的正确领导下，在市人大、市政协的依法监督和民主监督下，全面落实中央和省委稳增长、调结构、促改革、惠民生的各项政策，研究出台了促进经济稳定增长的26条意见，在逆境中奋进，在挑战中前行，全市经济走势趋稳向好、结构调整稳中有进、民生福祉持续改善。预计全年实现生产总值244.5亿元，同比增长7.5%，其中第一产业17亿元，增长5%；第二产业170亿元，增长7.8%；第三产业57.5亿元，增长7%。完成固定资产投资244亿元，增长21%；大口径财政收入48.5亿元，增长39.5%；一般公共预算收入17.99亿元，增长13.4%；社会消费品零售总额64.3亿元，增长12%；进出口贸易总额20亿美元，下降43%；城镇居民人均可支配收入26403元，增长11%；农民人均纯收入9882元，增长11.5%。除地区生产总值、规模以上工业增加值、固定资产投资、农民人均纯收入、进出口贸易总额等五项指标增速未完成年度目标任务外，其他各项指标均完成了七届人大三次会议确定的目标任务。

　　——项目建设成效显著。坚持把深入实施“3341”项目工程作为调整经济结构、夯实发展基础的主要抓手，进一步健全完善项目建设推进机制，谋划储备500万元以上项目420项，概算总投资1100亿元；实施招商引资项目316项，到位资金201亿元，同比增长10.8%；第二十届兰洽会签约项目开工率达到71.4%；向上争取项目资金23.3亿元，增长37%；确定实施的84个重点项目开工率79.8%。30万吨铜材深加工、330千伏河西堡变电站、2&times;330MW热电联产及供输水管线、太西煤金昌物流中心一期等项目投产投运，石羊河流域重点治理、东河灌区续建配套与节水改造、永昌县新增粮食产能等工程完成年度任务，“引大济西”工程规划报告通过水利部水规总院初评，兰州至张掖铁路新增三四线、交通路网工程、金昌铁路现代物流中心、金昌养老康复中心等重大项目前期工作积极推进。

　　——工业经济趋稳向好。认真落实市级领导包抓重点企业制度，不断强化“一企一策”服务措施，深入开展“抓管理、降成本、增效益”活动，对23户重点规模以上企业生产经营中的44个难点问题逐项研究解决，工业经济在断崖式下跌的困境中负重前行，逐步实现了止滑扭亏、由负转正、趋稳向好。预计全年完成规模以上工业增加值140亿元，同比增长7.6%，净增规模以上工业企业23家。10万吨无机纤维一期、300万只圆筒印花镍网扩建等项目建成投产，铜阳极泥贵金属综合回收利用、羰基镍及铂族金属原料制备、40万吨离子膜烧碱二期、30万吨PVC、16万吨干法乙炔、60万吨低温煤干馏、金川集团建设产业园、甘肃有色金属新材料创新创业示范园等项目加快推进，风电建成并网19.8万千瓦，光电建成并网180.5万千瓦，“一区三园”聚集功能显著增强，我市被列为全国有色金属新材料战略性新兴产业区域集聚发展试点和全省新能源电力就地消纳试点。

　　——“三农”工作稳步推进。现代农业稳步发展，完成高效农田节水53.75万亩，新改建食用菌棚1014座、日光温室1046座、标准化养殖小区(场)21个，认定家庭农场188个，新增市级农业产业化重点龙头企业15家、农民合作社303个，土地流转率49.6%，清河现代循环农业产业园被列为省级农业科技园区。农村基础条件持续改善，改造建设农村集中居住区25个、农村居民小区6个、公租房居民小区4个，改造新建农宅4168套，新改建维修渠道122.5公里、乡村道路62公里，行政村等级公路覆盖率达到100%。扶贫开发与“双联”行动融合推进，落实财政扶贫资金3503万元，实施了5个整村推进项目和35个扶贫项目，永昌县扶贫对象人均纯收入同比增加26.7%，贫困人口减少1万人。完成职业技能培训7282人，创业能力培训1572人，输转城乡富余劳动力8.06万人，实现劳务收入13.2亿元。

　　——第三产业发展提速。坚持把文化旅游产业作为第三产业加快发展的重要增长点，编制了文化产业发展规划，制定了促进旅游业加快发展的意见，出台了鼓励游客来金旅游优惠补贴暂行办法，启动了“紫金花城•神秘骊靬”大景区建设性规划编制工作，举办了第三届骊靬文化国际旅游节和中国•金昌国际青少年生存训练营活动，金昌文化街、金川区薰衣草种植示范基地、金水湖景区观赏园等项目建成，市图书馆基本建成，市博物馆、市传媒中心等项目主体完工，骊靬文化产业园、金昌华轩欢乐谷青少年生存夏令营等项目顺利实施，骊靬古城景区创建为AAAA级旅游景区。预计实现文化产业增加值2.4亿元，同比增长28%；旅游收入11.9亿元，增长29.5%。商贸物流业加快发展，金昌商会大厦、紫金广场等项目建成投用，国芳万和商城、永昌万祥城二期等项目建设进展顺利，改造完成村级商贸综合服务社19个，新建改造农贸市场4个。大力培育新型业态，有6家企业在“特色中国甘肃馆”上网交易，144户企业及商户在互联网上开设网店，22家中小企业特色产品应用电子商务营销。积极搭建银企融资平台，10家金融机构与207户企业签约信贷意向资金166.21亿元，为企业发放贷款86.59亿元，签约资金履约率达到52.1%。

　　——生态环保工作切实加强。坚持把生态文明建设放在突出地位，大力实施重点生态林业工程， 建立国家级保护性耕作项目核心示范区2个，完成人工造林2.25万亩，封山育林1.3万亩，治理沙化土地27万亩，新城子镇油菜花景观荣获2014年“中国美丽田园”称号。积极配合市人大呼吁加强祁连山水源涵养地保护工作，引起省人大和全国人大的重视关注，全国人大常委会办公厅召开代表建议办理工作座谈会，专题督办尽快制止在祁连山自然保护区采矿、探矿活动，遏制生态环境恶化的建议，水源涵养区采矿勘探行为得到遏制。启动大气污染治理计划，实施生态环保“十大工程”，治脏、治乱、治差、治污、治堵等“五治专项行动”成效明显，环保部综合督查组反馈的6项113个环保问题已整改86个，对查实的21起环境违法行为给予了行政处罚，责令停产整改企业14家，市区环境空气质量优良天数达到283天。

　　——城镇化步伐加快。编制完成市中心城区风貌及色彩规划和综合管网规划、龙首新区和高新产业园区控制性详细规划、市公共场所林荫停车场和29个行政村村庄整治以及4个省级美丽乡村建设等规划，永昌县被确定为全省“多规合一”示范县。市区道路新改建及绿化工作完成年度任务，市行政中心即将建成投用，盘旋路西南角城市棚户区改造、市限价商品房、市公安局业务技术用房、金昌海关及出入境检验检疫局办公楼、龙首湖景观工程等项目进展顺利，既有居住建筑供热计量节能改造加快推进，永昌县城供水改扩建工程开工建设，河西堡镇污水处理工程基本建成，美丽乡村示范点建设顺利推进，市区建成区绿地率33.1%，绿化覆盖率37.4%，人均占有公园绿地19.7平方米，我市被确定为国家新型城镇化综合试点市，永昌县和金川区双湾镇被确定为全省新型城镇化试点，城镇化率提高到66.8%。坚持创建为民、创建靠民、创建惠民，全体市民以加快建设繁荣文明和谐现代金昌、宜居宜业宜游幸福家园的生动实践，助推全国文明城市创建活动向纵深发展，我市顺利接受了中央文明委测评。

　　——社会事业全面进步。教育基础条件不断改善，冶金职业技术学院二期、理工中专迁建项目顺利实施，建成乡镇高标准中心幼儿园10个，义务教育薄弱学校改造建筑面积23.9万平方米，我市被确定为全省基本实现教育现代化建设实验区，金川区义务教育均衡发展通过国家级评估验收。科技创新步伐加快，授权专利410件，获批成立省级工程技术研究中心3家，分别有5家企业被认定为国家高新技术企业和省级专精特新中小企业，应用类科技成果转化率达到35%。医疗卫生基础条件不断完善，建成46所标准化村卫生室和7所乡镇卫生院附属用房，市人民医院和市第一人民医院迁建项目主体封顶，创建全国人口计生利益导向政策体系示范市通过评估验收。文化体育事业加快发展，建成农民工文化服务点21个、“乡村舞台”63个，完成“一村一场”建设项目51个、体育惠民工程6个、乡镇体育健身提升工程3个，我市代表团在省第十三届运动会上荣获团体总分第四名。深入开展安全生产大排查、矛盾纠纷大调处、交通安全大整顿、消防隐患大排除和社会治安大整治等社会治理“五大专项行动”，“平安金昌”建设全面推进，社会大局和谐稳定。统计、邮政、通信、人防、气象、档案、地方志、妇女儿童、防震减灾、民族宗教、外事侨务、住房公积金和食品药品安全等社会事业都取得了新进步。

　　——民生福祉持续改善。省列13件、市列10件民生实事全面落实。新增小额担保贷款1.76亿元、城镇就业2.6万人，城镇登记失业率控制在2.92%。居民医疗保险筹资标准由每人每年360元提高到400元，社会养老保险基础养老金由每人每月65元提高到75元，农村五保供养标准由每年4500元提高到5010元。城市低保标准由每人每月306元提高到458元，农村低保标准由每人每年1907元提高到2193元，新增城乡低保并轨乡镇4个。13076套保障性住房全部开工建设，主体竣工11546套，完成农村危旧房改造2000户。建成城市社区老年人日间照料中心4个、农村互助老人幸福院40个。给予特困残疾人和70岁以上一级残疾人每人每月100元的生活补贴，完成农村残疾人危房改造210户，帮助549名残疾人实现了就业和自主创业。

　　——改革开放力度加大。制定了市属国有及国有控股企业负责人经营业绩考核办法，金泥集团股份制改革和三洋金源农牧公司上市工作持续推进，金川集团公司总校及中小学校移交地方管理，八冶集团公司被列为全省发展混合所有制和员工持股试点企业。煤矿兼并重组工作积极推进，关闭了15对产能3万吨及以下的矿井。集体林权制度配套改革和水利改革试点等工作有序开展，金川区整区和永昌县南坝乡整乡推进土地确权试点登记工作全面完成。非公经济各项扶持政策全面落实，工商登记制度改革积极推进，中小企业公共服务平台建成营运，新增市场主体1857户、注册资本41.37亿元，分别同比增长28.71%、531.35%。财税体制改革取得实质性进展，“营改增”试点工作平稳有序推进，减负面达到26.24%。成功组建市交通投资公司和开发区投资公司，35家企业在甘肃股权交易中心实现托管，31家企业在甘肃股权交易中心挂牌交易。对外开放水平不断提高，向西开放步伐加快，新增外贸企业15家。

　　——政府效能明显提升。坚决贯彻中央八项规定精神和省市委相关规定，围绕反对形式主义、官僚主义、享乐主义和奢靡之风，扎实开展群众路线教育实践活动，大力开展“三进三帮”活动，政风建设取得积极成效。自觉接受人大法律监督、政协民主监督和社会公众监督，积极听取民主党派、工商联、无党派人士和人民团体意见，办理市人大代表意见建议118件、政协提案179件，办结率均达到100%。修订完善政府工作规则、政府法律专家咨询委员会工作规则和差旅费、会议费、培训费等管理办法，规范行政行为、规范权力运行、规范资金管理的制度体系初步形成。工商、质监移交地方管理，组建了市卫生和计划生育委员会，清理了市级41个部门的行政审批项目，保留271项，减幅40.7%。清理处置违规配备公务用车38辆，市级“三公”经费支出同比下降30%。严格落实党风廉政建设主体责任，扎实推进惩治和预防腐败体系建设，深入开展廉政风险防控工作，强化行政监察和审计监督，从源头上遏制了违纪违法案件的发生，树立了为民务实清廉的良好政府形象。

　　各位代表，过去一年，我市取得的成绩来之不易，这是市委贯彻中央和省委决策，统揽全局、正确领导的结果，是市人大、市政协有力监督、鼎力支持的结果，是地企、军地密切配合、通力协作的结果，是全市广大干部群众团结奋进、顽强拼搏的结果。在此，我代表市人民政府，向过去一年所有为金昌改革发展稳定作出贡献的各族各界人士，表示衷心的感谢！

　　在发展稳中有进、经济回升向好的同时，我们也更加清醒地认识到，稳中也有忧、进中也有险，金昌发展还面临着不少困难和问题：一是在全面认识和准确把握新常态的内涵、特征、表现形式方面还很不够，新常态下驾驭经济工作的能力和水平有待于进一步提升；二是经济总量较小，工业产品初级化、低端化特征明显，农村经济整体发展水平不高，第三产业发展滞后，非公经济规模偏小，调结构、转方式的措施不多、步伐不快；三是生铁、水泥、化工、有色金属等主导产品需求持续低迷、价格低位震荡，工业经济发展困难增多、风险加剧、压力加大；四是带动经济转型升级的重大项目储备不足，固定资产投资后续乏力，循环产业链条抵御市场风险能力弱；五是自主创新能力不强，企业技术研发能力不足，科技成果转化缓慢，创新驱动机制有待完善；六是资源环境和土地、资金等生产要素约束趋紧，节能减排和环境保护压力进一步加大；七是“四风”问题尚未根除，为官不为、庸官懒政、消极怠政现象还不同程度存在，政府执行力和公信力有待进一步增强，等等。我们一定要高度重视自身工作中存在的困难和问题，本着对党和人民高度负责的精神，采取更有创造性、更有针对性的措施认真加以解决，不辜负全市人民的期望！

　　二、2015年主要工作

　　各位代表，今年是全面深化改革的关键之年，是全面推进依法治国的开局之年，也是全面完成“十二五”规划的收官之年，做好今年的政府工作意义重大。

　　今年政府工作的总体思路是：以党的十八大和十八届三中、四中全会精神为指导，深入学习贯彻习近平总书记系列讲话精神，坚持稳中求进总基调，主动适应经济发展新常态，紧紧围绕全面建成小康社会目标，坚持发展第一要务，强化质量为先、生态为重、创新为魂、民生为本理念，重点推进结构调整、循环发展、城乡统筹、环境优化、治理创新，加快经济转型和城市转型，促进经济平稳发展和社会和谐稳定。

　　经济社会发展主要目标是：生产总值增长5.5%左右；规模以上工业增加值增长4%左右；固定资产投资增长6%左右；一般公共预算收入增长8%左右；社会消费品零售总额增长12%左右；进出口贸易总额增长7%左右；城镇居民人均可支配收入增长9%左右；农民人均纯收入增长11%左右；居民消费价格指数涨幅控制在3%以内；单位生产总值能耗下降2.5%以上；城镇化率提高1个百分点以上；城镇登记失业率控制在4%以内；人口自然增长率控制在6.5‰以内。

　　实现上述目标，需要全市上下勇于创新，奋力拼搏。必须以敢于啃硬骨头、敢于涉险滩、敢于过深水区的劲头，以改革的精神、思路、办法解决发展中的问题。必须认识新常态、适应新常态，按照市场要活、创新要实、政策要宽的要求，围绕新型工业化、信息化、城镇化、农业现代化，坚决调、果断转，加快把新产业、新业态、新模式、新产品培育发展成新的经济增长点。必须立足实际、加快发展，既不能片面追求粗放的高增长，也还要保持合理发展速度防止经济惯性下滑；既要保增长，更要保就业，努力让科学发展的步子更好更快、改革创新的步伐有力有序。必须生态优先、保护环境，始终把生态文明建设融入经济社会发展的各领域，进一步形成尊重自然、顺应自然、保护自然的良好氛围，建设地绿水净、天朗气清、惠风和畅的美丽家园。必须以人为本、改善民生，突出保障基本民生、底线民生、热点民生，切实提升基本公共服务均等化水平，让全体市民过上更加美好的幸福生活。必须准确把握政策，积极转化政策，大力开展“政策落实年”活动，引导全市上下真正把精力用在研究政策上、把智慧用在谋划项目上、把功夫用在推动落实上、把心思用在为民谋利上。必须提振精气神、汇聚正能量，以“三严三实”的过硬作风不断克服前进道路上出现的各种艰难险阻，使各项工作目标精确、对策精准、效果精致。

　　围绕以上预期目标，主要做好六个方面工作。

　　 (一)积极调整经济结构，切实提高经济发展质量和效益。坚持存量调整与增量提质相结合，牢牢把握基础设施互联互通和新技术、新产品、新业态、新商业模式投资方向，深入实施“3341”项目工程，更好发挥投资在经济发展中的关键作用，形成多点支撑、多元发展、多级突破的产业格局。

　　推动工业转型升级。工业是我市经济发展的脊梁和支柱，是财税收入的主要来源，必须深入实施“工业强市”战略，围绕发展壮大“一区三园”，坚持走战略性新兴产业培育、循环产业链条延伸、传统优势产业改造升级“三位一体”的工业经济转型升级道路，积聚新动能，激发新活力，促进产业向高端化转变，产品向终端化延伸。大力发展有色金属新材料产业，围绕推进金昌有色金属新材料战略性新兴产业区域集聚发展试点工作，积极支持金川集团公司重点发展铜及铜合金加工材、镍及镍合金加工材、金属盐类、电池材料、粉体材料及制品、电线电缆、高纯金属、贵金属材料等“八大有色金属新材料产业”，切实抓好高目数圆筒印花镍网、高能辐照交联电缆、单晶铜键合引线等项目建设，开工建设1万吨碳酸锂加工、超细金属及氧化物试验生产线、10万吨无机纤维二期、高端装备特种合金材料等项目，积极做好软通动力金昌大型数据中心和有色金属交易平台等项目前期工作。大力发展工业循环经济，围绕丰富发展循环经济“金昌模式”，立足已经形成的工业循环产业基础，不断延伸产业链条，壮大产业集群，促进化工产业向精细方向发展。重点实施2万吨硼酸及资源循环利用、瓮福硫磷化工产业园等一批循环化项目，40万吨离子膜烧碱二期、30万吨PVC等项目建成投产，促进全社会领域循环，建设覆盖全市的资源循环利用体系，推动国家循环经济示范市和全国低碳试点城市建设。大力发展新能源产业，围绕建设全国新能源示范城市，着力推进风光电项目建设，切实抓好艾力克光伏发电组件、110千伏龙首变增容改造、以及西大河北总干渠一、二级水电站、东河湾三级水电站等项目建设。积极开展新能源电力就地消纳试点工作，不断降低企业生产成本，促进新能源产业良性发展。

　　加快现代农业发展。坚持走品种特色化、基地规模化、生产标准化、经营产业化“四化共进”的现代农业转型升级道路，推动农业发展由数量增长为主转到数量质量效益并重上来，由依靠资源和物质投入转到依靠科技进步和提高劳动者素质上来。突出特色，提升产业竞争力。进一步优化种养业结构，引导农民以市场为导向，发展适销对路的优质特色农产品，大力发展品牌农业、精品农业、生态休闲观光农业，重点扶持发展优质肉羊、优质肉牛、无公害蔬菜、现代制种、食用菌、中药材、优质牧草等特色农产品基地规模，大力实施舍饲养殖能力提升计划和百村万棚质量提升计划，规范发展标准化养殖小区(场)20个，新改建日光温室、食用菌棚1000座，新增塑料大棚3000亩，特色优势农作物种植面积达到95%以上。扶优助强，提升市场带动力。按照基地型、集团化、链条式思路，切实把产业链、价值链等现代产业发展理念和组织方式引入农业，延伸产业链、打造供应链、形成全产业链，让农民从产业链中获得更多利益。高度重视涉农政策和农副产品信息的收集、研判和发布，推进家庭经营、集体经营、合作经营、企业经营等多元化新型农业经营主体共同发展，实施农垦天牧乳业、弘顺肉制品加工、三强啤酒麦芽生产线扩建以及2万吨生物降解母料及制品加工、5000吨农产品恒温库等农业产业化项目，培育示范型家庭农场20家、农民合作社30家，培训农产品经纪人270人，促进一、二、三产业融合互动。固本强基，提升综合生产力。着力提高现代农业装备水平，做好石羊河流域重点治理项目收尾工作，继续实施永昌县新增粮食产能工程、中央财政小型农田水利高效节水灌溉示范、高标准农田建设等项目，争取开工建设西河灌区续建配套与节水改造、金川河工农渠首病险水闸除险加固、西坡光伏产业园区防洪等项目，全力推进“引大济西”等项目前期工作，推广农田节水38万亩，建立农业科技示范点25个、示范面积10万亩，引进示范农业新品种、新技术120个，农机化综合作业水平达到80%。

　　推动第三产业提质增效。坚持走文化旅游业融合引领、现代服务业多元主导、传统服务业改造提升等“多业并举”的第三产业转型升级道路，促进第三产业发展提速、比重提高、质量提升。突出发展文化旅游业，按照景城一体化建设理念，围绕打造“中国镍都、神秘骊靬、紫金花城、沙海乐园”四大形象品牌，加快理顺旅游管理体制，加大财政支持力度，全面启动“紫金花城•神秘骊靬”大景区建设，重点实施紫金花城景观提升改造、骊靬文化产业园、金川民俗文化街、青少年生存训练营、镍都工业游等旅游项目，认真编排体现地方文化特色的舞台剧，加快发展融现代文明、田园风光、乡村风情于一体的“农家乐”、“乡村旅馆”等新业态，力争金水湖景区创建为AAAA级旅游景区，文化产业增加值同比增长25%，旅游收入增长22%。大力发展商贸物流业，积极推广现代物流方式，加强流通基础设施建设，开工建设镍都商业大厦改造、金三角市场改造等项目，积极开展金昌铁路现代物流中心、甘电投金昌发电公司铁路专用线和电煤应急储备中心等项目前期工作，培育新的增长动力。加快发展新兴服务业，积极适应以个性化、多样化渐成主流的消费新模式，推进城域光网建设，实现城市光纤进楼入户和全市100%的行政村通宽带。着力促进大数据、物联网、互联网等与现代制造业、特色农业相结合，推动电子商务、网络信息、现代物流业和互联网金融等产业融合发展，着力开拓中介服务、文化教育、网络购物、家居服务、高新技术服务等新领域，积极开发香草花卉观赏体验、中草药养生保健等新业态，使消费在经济发展中更好地发挥基础作用。

　　 (二)全面深化改革，切实激发经济增长动力和活力。全面落实各项改革部署，使市场在资源配置中起决定性作用和更好发挥政府作用，最大限度激活市场主体，增强发展活力。

　　深化国资国企改革。全面贯彻落实“甘肃国资国企改革23条”，抓紧出台我市具体实施意见，牢牢把握出资人定位，准确界定经营性国有资产范围，全力推动经营性国有资产集中统一监管，实现由管资产向管资本转变。大力支持八冶建设集团公司发展混合所有制和员工持股试点改革，积极支持金川集团公司全面放开有色金属深加工领域，着力支持金化集团、金铁集团、金泥集团引进战略性合作伙伴，发展混合所有制经济。加快推进三洋金源农牧股份公司、金泥集团公司、宇恒镍网公司等企业上市步伐，引导非公企业通过产权合并、股权收购、资产置换、债务重组等形式参与国有企业改制重组，着力化解国有企业不良资产，促进各种所有制经济取长补短、相互促进、共同发展。深化企业领导人员管理体制机制改革，加快企业领导人员去“行政化”步伐，加速建立有别于党政领导干部、符合市场化要求的企业领导人员选用新机制，更好地发挥企业家作用。抓紧建立与经营管理业绩和风险责任紧密挂钩、与企业领导人员分类管理相适应、与选任方式相匹配的国企管理人员薪酬分配制度，实现薪酬水平相当、结构合理、管理规范、监督有效。创造更加公平的市场环境、政策环境和社会环境，使生产小型化、智能化、专业化为产业组织新特征的新兴产业、服务业、小微企业的作用更加凸显。

　　深化农村综合改革。抓紧建立县级农村产权交易市场，全面完成金川区全国农村改革试验区工作任务，加快农村承包地、宅基地和农宅、农牧业设施、草原、集体建设用地确权登记颁证工作，赋予农民对承包地的占有、使用、收益、流转及承包经营权的抵押、担保权能，促进农村产权公开规范交易流转。着力推广金川区“3331”产业分工模式，积极探索推广土地互换并地和经营权入股、土地托管等流转方式，引导农业企业采取保底收购、股份分红、利润返还等方式，与农户建立紧密的利益联结机制，落实土地所有权，稳定农户承包权，放活土地经营权，发展多种形式适度规模经营。加快推进供销社改革，创新供销社组织体系，推进城乡流通现代化建设，畅通农产品流通渠道，切实把供销合作社打造成为农民生产生活服务的生力军和综合平台。

　　深化财税金融改革。认真贯彻实施新的《预算法》，完善政府预算体系，改进预算控制方式，健全透明预算制度。清理甄别政府存量债务，促进政府债务分门别类纳入全口径预算管理。切实加强政策研究和转化工作，进一步加大向上争取政策和项目资金力度，力争向上争取项目资金25亿元以上，同比增长14.5%。清理规范各类财税优惠政策，全面完成“营改增”任务，促进全民创业，推动小微企业加快发展。鼓励各类资本参与金融机构增资扩股，支持农村信用联社改组成立农村商业银行，加快组建村镇银行、资金互助社。大力引进创业投资基金，积极探索建立产业发展基金，全力激活民营资本，筹建旅游投资公司和水利投资公司，扶持壮大市建设投资公司、市交通投资公司、开发区投资公司和小额贷款公司、融资性担保公司等金融机构融资规模，优化信贷结构，推进资源、资产、资本一体化运作。

　　扩大对外开放合作。紧紧抓住建设“丝绸之路经济带”的战略机遇，创新开放模式，拓展开放领域，集成开放优势，支持金川集团公司、八冶建设集团公司等企业在境外投资设厂，建立生产基地、原材料基地和营销网络，加快向西向北开放步伐。准确把握新常态下产业发展方向和产业转移趋势，统筹引资、引技和引智，推进精准招商和产业链招商，利用各种节会，借助各类平台，吸引外企、央企、民企聚焦，重点引进新兴产业高端项目以及产业链、价值链高端环节和缺失环节，确保招商引资到位资金达到245亿元以上。启动金昌海关及出入境检验检疫局报关报检业务，支持开发区探索与发达地区友好开发区开展“园中园”合作开发模式，加强与意大利博拉等城市交流合作，努力在开发开放中寻找、发掘和引导、培育新的经济动力。

　　 (三)加强生态文明建设和环境保护，切实推动绿色循环低碳发展。顺应人民群众对良好生态环境的期待，坚持源头严防、过程严管、后果严惩，治标治本多管齐下，坚决不碰生态红线，坚决严守生态底线，努力使金昌天更蓝、水更清、空气更清新、环境更宜人。

　　加强生态建设。抢抓国家加快生态文明建设和省上打造生态战略平台的历史机遇，按照“南护水源、中建绿洲、北治风沙”的总体思路，大力推进以“增核、扩绿、联网”为主要内容的戈壁绿城建设，重点实施退耕还林、退牧还草、天然林保护、“三北五期”和金川国家级矿山公园等项目，积极申报规划建设北海子国家湿地公园，加快推进祁连山冰川与水源涵养生态功能区生态保护与建设项目前期工作，开工建设金川区沙化土地封禁保护补助试点、“小渊基金”援助防沙治沙示范林、造林补贴试点、优质种苗繁育基地、灰枣基地、高速公路东出口枢纽区绿化等项目，加快风光电场生态修复等项目，完成人工造林面积1.75万亩、封山育林3.1万亩、抚育中幼龄林1.6万亩、沙化土地综合治理15万亩、义务植树80万株。认真做好市区拓建道路绿化、金川河河道治理绿化、新建居民小区绿化、市区北部绿色长廊生态环岛休闲景区建设等项目，龙泉景观带三期、龙首湖公园等景观工程建成投用。

　　推动节能减排。严格贯彻落实新《环保法》，坚持把节能减排作为硬任务、硬指标、硬约束，促进结构减排、工程减排、管理减排和建筑节能共同推进，促进经济向低污染、低能耗、高附加值的产业链高端迈进，继续着力抓好环保部西北综合督查组反馈问题的治理整改，重点抓好金川集团公司冶炼烟气深度治理、热电一车间环保技术改造、金化集团公司锅炉脱硫改造等重大环保工程，确保二氧化硫、氮氧化物、化学需氧量、氨氮等主要污染物完成减排任务。加强建筑施工扬尘、露天烧烤餐饮油烟、垃圾焚烧、秸秆焚烧、机动车尾气等大气污染防治，强化对重点排污企业的监管，探索合同能源管理、碳排放交易、排污权交易等新机制新模式。加快实施开发区工业污水处理、市污水处理升级改造等项目，全面推进公共机构和全民节能行动，既有居住建筑热计量及节能改造10万平方米。狠抓资源节约集约利用，积极推广清洁能源，大力推广清洁生产技术，切实减轻环境压力。

　　推进固废利用。围绕建设全国工业固废综合利用示范基地，重点实施冶炼含铜废渣废酸资源化利用、镍铜矿伴生铂族贵金属高效回收与综合利用等项目，开工建设金川神雾80万吨铜渣尾矿处理、电石炉净化尾气综合利用、报废汽车回收拆解扩能改造以及城市建筑垃圾处理循环再利用等项目，京天福磷石膏综合利用等项目建成投产，工业固体废物处置利用率达到30%以上。大力发展节水、绿色、生态、立体、循环生态农业，秸秆综合利用率达到82%，废旧农膜回收利用率达到72%，规模畜禽养殖废弃物利用率达到87%，做好清河现代循环农业产业园创建国家级循环农业示范园申报工作，把金川循环农业综合示范园、金昌现代畜牧循环产业园创建成省级循环农业示范园。

　　 (四)强化城镇规划建设管理，切实推进新型城镇化进程。以建设繁荣文明和谐现代金昌、宜居宜业宜游幸福家园为目标，全面推进国家新型城镇化综合试点和永昌县、金川区双湾镇省级新型城镇化试点工作，完善城镇功能，提升城镇品位，彰显城镇特色。

　　科学定位，高起点规划。坚持中心带动与多点支撑并举，城区都市化与农村城镇化并重，顺应发展型迁移为主的农村劳动力转移形势，统筹产业、就业、环境和聚集人口，进一步优化空间布局，完善载体功能，美化市容环境，突出城镇特色，促进农业转移人口市民化、外来人员深度融合本地化、失地农民就地就业城镇化。重点编制完成新型城镇化规划、城镇体系规划、市区北部绿色生态景观工程规划、市区重点地段和重点建筑单体方案规划、永昌县城“多规合一”规划，同步编制重点乡镇、村庄整治、美丽乡村、农村集中居住点等规划，不断提高规划管理水平。立足农村生产发展、生活方式转变、文明乡风形成、村容村貌整治，进一步提升农宅设计标准，打造富有特色、个性鲜明的建筑风格。健全完善城乡规划编制审查、公示、听证和监督制度，维护规划的严肃性和权威性。

　　以人为本，高标准建设。坚持设施配套、产城融合，加快完善吸引人、留住人、造福人的城镇功能，努力使居民都能安其居、畅其行、乐其业。加快推进新区建设和老城区改造，突出抓好龙首新区限价商品房、龙首壹号住宅小区、金沙公馆住宅小区、龙庭佳苑住宅小区、“印象金昌”文化创意园商业风情街、国芳万和商城等项目建设，市传媒中心、市人民医院和市第一人民医院迁建、市公安局业务技术用房、市博物馆等项目建成投运。加快实施永昌至红沙岗一级公路改建等项目，新建及改造城市道路5条，金阿高速公路建成通车。着力推进兰州至张掖铁路新增三四线、金山高等级公路、城市应急水源工程等项目前期工作，加快永昌县城供水供热及污水处理中水利用和河西堡镇镇区面貌三年大变样工程进程，形成规模适度、功能完善、布局合理、宜业宜居的特色城镇体系。按照城中村融入城市、近郊村服务城市、远郊村依托城市、偏远村相对集中居住的路径，稳步推进新农村建设，切实抓好城中村改造，着力加强农村集中居住点基础设施和公共服务配套设施建设，保护青山绿水，打造田园风光。加快改善人居环境，提高农民素质，推动“物的新农村”和“人的新农村”建设齐头并进。

　　创新机制，高水平管理。坚持把法治作为治理城市的基本方式，着力推行精细化、人性化和智能化的城市服务管理方式，努力使城市因人更具活力、人因城市更加幸福。积极探索建立比较完善的农业转移人口市民化成本分担机制、多元化可持续的城镇化投融资机制、农村闲置宅基地市场化流转退出机制，为全省提供可复制、可推广的经验和模式。深入开展国家环保模范城市、国家“智慧城市”创建工作，积极开展健康城市建设工作，全面做好国家卫生城市复审工作，持续开展治脏、治乱、治差、治污、治堵等“五治专项行动”，加快“数字城管”二期项目建设，综合整治城乡结合部、棚户区、背街小巷、集贸市场，加大对城市规划区内违法建筑查处拆除力度，努力让城市更加宜居、社会更加有序、生活更加便利。深入开展社会主义核心价值观宣传教育，积极推进社会诚信体系建设，扎实开展群众性精神文明创建活动，着力提升市民文明素质，培育城市人文精神，增强市民的自豪感和城市管理的内在动力。

　　 (五)统筹发展各项社会事业，切实促进社会公平正义。优化财政支出结构，提高基本公共服务均等化水平，促进社会公平正义,进一步提高城乡居民幸福感与社会和谐度。

　　加快脱贫致富步伐。把“双联”行动同“1236”扶贫攻坚行动结合起来，坚持统筹扶贫、精准扶贫、生态扶贫相结合，着力帮助贫困村、贫困户拓宽致富思路，培育富民产业，广辟增收渠道，确保贫困村农民人均纯收入增长幅度高于全市2个百分点以上。重点帮助谋划一批基础性、公共性、民生性建设项目，加快通村道路、村内道路硬化和安全饮水工程建设，着力实施石羊河流域重点治理生态移民和红光园艺场易地扶贫搬迁项目。

　　增强科技创新能力。强化企业创新主体地位，引导创新资源向企业集聚，打造创新团队、企业研发中心、协同创新平台，加快甘肃有色金属新材料创新创业示范园建设，开发区科技孵化和检测中心建成投运，促进创新成果资本化、产业化。深入实施“人才特区”工程，加快培养高层次创新创业人才、高水平管理人才、高技能实用人才，引进集聚更多领军型人才，支持大众创业，鼓励万众创新，使经济增长更多依靠人力资本质量和技术进步。

　　办好人民满意教育。实施义务教育改薄工程和第二期学前教育三年行动计划，每个乡镇办好一所中心幼儿园，扩大覆盖范围，完善关爱体系。开工建设市五中等项目，完成永昌县县域义务教育均衡发展国家级评估验收，缩小城乡、校际差距，破解择校难题。完成理工中专迁建项目，加快金昌职教城建设步伐，着力推进全省教育现代化试验区建设。

　　促进文化卫生体育事业发展。进一步完善公共文化服务体系，健全文化管理体制，鼓励社会力量、社会资本参与公共文化服务。以“人才下沉、资源下沉”为突破口，积极推进公立医院改革试点工作，加快培养社区卫生服务中心全科医生队伍，加大乡村医疗队伍建设，促进基层医疗卫生机构提升服务能力和服务效率。加快市妇幼保健计生服务中心综合业务楼、市消毒供应中心等项目建设进度，开工建设金昌养老康复中心、市残疾人康复中心等项目。改造提升市体育场馆，完善公共体育设施，举办金昌市第七届运动会，加快发展体育产业，促进体育消费。

　　加强和创新社会治理。坚持系统治理、依法治理、综合治理、源头治理，继续开展社会治理“五大专项行动”，深入推进“平安金昌”建设，健全完善社会治安防控体系，净化社会治安环境，依法打击各种违法犯罪，健全重大决策社会稳定风险评估机制、矛盾纠纷化解机制、群众权益保障机制和基层维稳应急处置机制，实现政府治理和社会自我调节、居民自治良性互动。健全基层综合服务管理平台，加强应急指挥平台建设，有效应对各类突发公共事件。落实最严谨的标准、最严格的监管、最严厉的处罚、最严肃的问责，保障食品药品安全，开工建设集中生产、统一配送、全程追溯的金川区食品工业园区。加快推动传统媒体和新媒体融合发展，做好网络管理和舆论引导工作，弘扬主旋律，传播正能量。深入推进民族团结进步及和谐寺观教堂创建工作，促进各族群众和睦相处、和衷共济、和谐发展。积极创建双拥模范城“七连冠”，加强军政军民团结，促进军民融合式发展。

　　 (六)进一步保障和改善民生，切实增进人民福祉。坚持守住底线、突出重点、完善制度、引导舆论的基本思路，更加关注民生、切实保障民生、不断改善民生，在努力完成省上下达我市为民实事任务的基础上，实施民生“八大工程”，进一步织好民生保障安全网。

　　就业增收工程。新增城镇就业1万人以上。新增小额担保贷款1亿元。输转城乡富余劳动力8万人，实现劳务收入11亿元。

　　社会保障工程。城乡居民医疗保险补助标准提高12.5%，住院实际报销比例达到65%。城乡居民基本养老保险基础养老金由每月75元提高到80元。新建农村互助老人幸福院30个、城市社区日间照料中心3个。城乡低保标准提高15%，完成水源镇、新城子镇、六坝乡、红山窑乡4个乡镇城乡低保并轨任务。

　　素质提升工程。城乡就业技能培训6000人，创业能力培训1000人。农业科技人才培训2400人。完成“改善义务教育薄弱学校基本办学条件”项目校舍建设12.7万平方米。资助家庭贫困学生5500人。

　　全民健康工程。建立生活饮用水在线监督监测预警系统，设置监测点4个(市区2个、河西堡镇1个、金川公司1个)。在金川区设立地下水水质检测点，促进地下水资源有效保护。建成乡镇及社区体育健身中心建设项目2个，完成“一村一场”建设项目10个。推进文化惠民工程建设，建设“乡村舞台”50个。

　　安居宜居工程。新建公租房2320套、棚户区改造安置房7539套。新增发放低收入住房保障家庭租赁补贴1300户。推进美丽乡村示范村项目建设，建设美丽乡村4个，每个乡镇至少规划新建改造或扩建1个农村集中居住点。新改建农宅500套。实施农村危房改造2000户，其中农村贫困残疾人危房改造210户。

　　畅通出行工程。继续推进金川区城乡客运站和永昌县客运中心项目建设，开工建设永昌县清河客运站。新改建农村公路50公里。新增公交车43辆、更新42辆，新增出租车260辆、更新240辆。

　　安全保障工程。完成“三品一标”示范基地10万亩。完成市级农产品质量安全检测中心建设，农产品质量抽检年平均合格率达到98%以上。完成县乡农贸市场改造建设项目2个，创建星级农家乐10家，改造村级商贸综合服务社10家，规范完善农资农副连锁示范店10家，新建农村合作经济组织10个。建成中心乡(镇)配送中心2个、乡(镇)超市7个，新建和改造村级综合服务社、农家店和便民店18个。加快推广应用食品药品安全追溯信息平台，电子监管率达到40%以上。新增放心粮油示范网点20个。

　　扶贫解困工程。在永昌县实施5个扶贫开发“整村推进”项目。在永昌县贫困村实施一批群众认可并广泛参与的富民产业、基础设施、困难家庭“两后生”培训等专项扶贫开发项目。

　　三、建设法治政府

　　认真贯彻落实党的十八届四中全会精神，牢固树立底线思维和法治思维，加快建设职能科学、权责法定、执法严明、公开公正、廉洁高效、守法诚信的法治政府。

　　依法确定职权边界。按照“法无授权不可为、法定职责必须为”的要求，坚持放管结合，科学合理调整机构设置，优化部门职责配置，进一步取消调整和下放市级行政审批事项，减少投资、生产经营活动、资质资格等方面的行政许可，坚决用政府权力的减法换取市场活力、社会活力的加法，使政府工作人员做到不触碰法律禁止的红线，不逾越法律禁止的底线，让政府一切工作都在法律框架内运行。加快建立权力清单制度，明确权力运行的程序、环节、过程、责任，严格依照法定权限和程序行使权力、履行职责，促进政府事权规范化、法律化。

　　依法健全决策机制。加强对政府工作人员在新常态下做好经济工作的理论学习，自觉接受市委的领导，自觉接受人大、政协、社会各界的工作监督、法律监督、民主监督和舆论监督，建立行政机关内部重大决策合法性审查机制、重大决策终身责任追究制度及责任倒查机制，履行公众参与、专家论证、风险评估、合法性审查和集体讨论等程序，做到政府重大改革、重大决策于法有据、程序正当、过程公开、责任明确。建立专家咨询制度，推进行政决策民主化、科学化和法制化，重点规范规划编制程序，健全规划衔接、论证、发布和评估制度，重点编制完成全市国民经济和社会发展“十三五”规划纲要和专项规划。

　　依法规范执法行为。以食药安全、工商质监、安全生产、资源环境、农林水利、交通运输、城乡建设、城市管理等领域为重点，进一步完善综合执法机制，切实解决权责交叉、多头执法和“临时工”执法顶责等问题。不断完善行政裁量权基准和执法全过程记录制度，健全评议考核、责任追究、持证上岗和资格管理制度，进一步提升执法效率和规范化水平。各级领导干部和全体政府工作人员要认真学习宪法和法律，尊崇宪法和法律，遵守宪法和法律，自觉在宪法和法律的范围内活动，绝不允许以言代法、以权压法、徇私枉法，自觉依法办事和依法行政。强化公民普法教育，完成“六五”普法工作，努力使全体公民都成为法治的忠实崇尚者、自觉遵守者、坚定捍卫者。

　　依法建设清廉政府。认真落实党风廉政建设主体责任，规范行政权力运行，加大对违规行为的行政问责力度，让干部学会在约束中工作、习惯在监督下干事。加快健全完善公共资源电子交易平台，加强对重点领域、重点项目、重点资金的监督检查，切实让暗箱操作没有空间，让腐败分子无处藏身，形成不敢腐的惩戒机制、不想腐的教育机制、不易腐的保障机制、不能腐的防范机制。大力弘扬艰苦奋斗、勤俭节约的优良传统，严格财政预算管理，确保行政运行经费零增长、“三公”经费负增长，政府自觉过“紧日子”，努力让群众过上“好日子”。

　　依法加强效能监察。巩固党的群众路线教育实践活动成果，坚决整治“慵懒散慢”、“门好进、脸好看、事难办”等问题，以踏石留印、抓铁有痕的作风抓落实、促发展，以政府工作效率的提高促进发展质量效益的提升。遵循公开为常态、不公开为例外的原则，加快电子政务建设，重点推进财政预算、公共资源配置、生态环境保护、重大建设项目批准和实施、社会公益事业建设告示领域的政府信息公开，让群众看得到、听得懂、信得过、能监督。进一步强化网络问政工作，及时回应群众关切，有效解决群众诉求。

　　各位代表，建设美丽金昌的光荣使命鼓舞我们砥砺奋进，全市人民的信任期盼鞭策我们奋勇前行。让我们在市委的坚强领导下，抢抓机遇、深化改革，齐心协力、埋头苦干，为共同谱写中国梦的金昌新篇章而努力奋斗！
